# Transcriptional profiling and muscle cross‐section analysis reveal signs of ischemia reperfusion injury following total knee arthroplasty with tourniquet

**DOI:** 10.14814/phy2.12671

**Published:** 2016-01-05

**Authors:** Jonathan B. Muyskens, Austin D. Hocker, Douglas W. Turnbull, Steven N. Shah, Brick A. Lantz, Brian A. Jewett, Hans C. Dreyer

**Affiliations:** ^1^ Department of Human PhysiologyUniversity of OregonEugeneOregon; ^2^ Genomics and Cell Characterization FacilityUniversity of OregonEugeneOregon; ^3^ Slocum Center for Orthopedics and Sports MedicineEugeneOregon

**Keywords:** Aging, clinical, hypoxia, NextSeq, stress, surgery

## Abstract

Total knee arthroplasty (TKA) is the most common and cost‐effective treatment for older adults with long‐standing osteoarthritis. Projections indicate that nearly 3.5 million older adults will undergo this procedure annually by the year 2030. Thus, understanding the factors that lead to optimal outcomes is of great clinical interest. In the majority of cases, tourniquet is applied during surgery to maintain a clear surgical field, however, there is debate as to whether this intervention is completely benign. In particular, muscle atrophy is a significant factor in preventing full functional recovery following surgery, and some evidence suggests that tourniquet application and the associated ischemia–reperfusion injury that results contributes to muscle atrophy. For this reason, we examined tissue level changes in muscle in TKA patients following surgery and found that there was a significant increase in cross‐sectional area of muscle fibers of all types. Furthermore, to detect changes not evident at the tissue level, we performed NextSeq analysis to assess the transcriptional landscape of quadriceps muscle cells following TKA with tourniquet and found 72 genes that were significantly upregulated. A large proportion of those genes regulate cell stress pathways, suggesting that muscle cells in our cohort of older adults were capable of mounting a significant response to cell stress. Furthermore, factors related to complement were upregulated, suggesting tourniquet may play a role in priming cells to ischemia reperfusion injury. Therefore, our analysis reveals potential harms of tourniquet during TKA, thus suggesting that surgeons should consider limiting its use.

## Introduction

Osteoarthritis (OA) of the knee is the leading cause of hospitalization for adults ages 45–84 years in the U.S. (Pfuntner et al. 2013), and 60% of adults over the age of 65 have OA (Parsley et al. 2010). Surgical remediation of the chronic knee pain caused by OA is successfully accomplished with total knee arthroplasty (TKA). In fact, in a sampling of Medicare beneficiaries (*N* = 124,986), OA is the leading diagnosis for 94% of all patients having TKA in the U.S. (Mahomed et al. 2005). In the U.S., in 2008, more than 650,000 TKAs were performed at a cost of $9 billion (Kurtz et al. 2011; Cram et al. 2012). This financial burden will only become more significant as the population of older adults increases. In fact, the number of TKAs is projected to reach 3.5 million surgeries performed in the U.S. annually by 2030 (Kurtz et al. 2011). Thus, improving the success of TKA is of utmost importance both for improving quality of care and reducing costs. Despite the incredible success of TKA in eliminating knee pain due to OA, postoperative recovery is often compromised by persistent muscle atrophy and reduced functional mobility. Quadriceps atrophy is responsible for the majority of functional deficit 1–3 years post‐TKA (Meier et al. 2009) by impairing balance (Moxley Scarborough et al. 1999), reducing mobility (Brown et al. 1995; Mizner et al. 2005c), and increasing fall risk (Moreland et al. 2004). Furthermore, patients with substantial quadriceps atrophy will find it difficult to exercise and will require significantly more physical therapy.

A tourniquet is routinely used during TKA to control blood loss, maintain a clear surgical field, and facilitate proper bone‐implant cementing. Results from a 2009 survey found that 95% of orthopedic surgeons use a tourniquet in those patients cleared of vascular disease (Berry and Bozic 2010). Before tourniquet application, the leg is elevated and an Esmarch bandage is applied in a distal to proximal fashion to exsanguinate blood from the operative limb. The effects of ischemia followed by reperfusion (I/R) on human skeletal muscle metabolism is poorly understood at the cellular level, however, there have been studies that suggest that there are potential clinically meaningful issues associated with tourniquet use. These issues include greater postoperative pain (Chen et al. 2014; Ejaz et al. 2014), reduced range of motion (Vandenbussche et al. 2002; Chen et al. 2014; Ejaz et al. 2014), and greater postoperative edema (Konrad et al. 2005; Chen et al. 2014). Further, there are reports that TKA performed without a tourniquet results in faster patient recovery (Ejaz et al. 2014), and a recently completed randomized trial found that patients in the tourniquet group had reduced strength recovery as compared to patients in the nontourniquet group at 3‐week and 3‐month follow‐up (Dennis et al. 2016).

In an attempt to gain a better understanding of the changes in skeletal muscle occurring after TKA at the molecular and cellular level, we have collected muscle biopsy samples before tourniquet inflation (baseline). In particular, we have measured a decrease in anabolic signaling (Ratchford et al. 2012), an upregulation of catabolic and cell stress pathways (Bailey et al. 2012), and induction of the unfolded protein response (UPR) due to endoplasmic reticulum (ER) stress (Hocker et al. 2013). Our working hypothesis is that the tourniquet‐induced oxygen deficiency that occurs during TKA may disrupt cell and tissue metabolism and potentially contribute to the rapid loss of muscle (1% per day) measured within the first 2 weeks after surgery (Dreyer et al. 2013). The objective of this study, therefore, was to measure the physical alterations of muscle cell (swelling) and subject biopsy samples to genomic sequencing in order to identify key regulatory pathways that are differentially expressed.

## Materials and Methods

### Ethics approval

This study was approved by the PeaceHealth Institutional Review Board, Sacred Heart Medical Center, at RiverBend and the Institutional Review Board, Research Compliance Services, University of Oregon and conducted in accordance with the Declaration of Helsinki. All subjects gave informed written consent prior to study participation.

### Subjects

We studied 13 subjects (nine females and four males) recruited from a pool of surgical candidates at the Slocum Center for Orthopedics and Sports Medicine. Subjects were between 60 and 78 years of age and were scheduled to have primary TKA. Exclusion criteria included untreated endocrine disease, significant heart, kidney, liver, blood or respiratory disease, vascular diseases, cancer, treatment with anabolic steroids or corticosteroids for greater than 1 week, and alcohol or drug abuse.

### Study design

Details of the study design have been published previously (Bailey et al. 2012; Ratchford et al. 2012; Hocker et al. 2013). Study subjects were admitted to the Sacred Heart Medical Center at Riverbend in a fasted state on the morning of surgery. Anesthesia was administered using standard methods as previously described (Bailey et al. 2012; Ratchford et al. 2012; Hocker et al. 2013). A 10‐cm wide Zimmer tourniquet was placed around the proximal third of the thigh and was not inflated. Prior to surgery, a biopsy of the vastus lateralis muscle on the operative leg was taken using a 5‐mm Bergstrom biopsy needle with applied suction. Following the first biopsy, the tourniquet was inflated to 300 mmHg or greater, depending on systolic blood pressure to ensure minimal blood flow to the operative leg. Average time of tourniquet application for all subjects was 40.2 min (SD = 5.1 min, range 33–50 min). Following surgery, a second biopsy was performed as close to two hours after tourniquet deflation as possible (average time = 139.8 min, SD = 33.1, range = 84–184 min).

### RNA preparation

Isolation was performed as previously described (Hocker et al. 2013) with modifications. Tissue samples were frozen in liquid nitrogen and stored at −80°C until use. A quantity of 5–20 mg of tissue was weighed and homogenized in 700 *μ*L Qiazol (Qiagen, Germantown, MD) on ice using a Heidolph Silent Crusher (Schwabach, Germany). Nucleic acid extraction was performed using 140 *μ*L of chloroform, the resulting aqueous layer was precipitated in 0.5 mL of isopropanol, and the resulting pellet was rinsed in ethanol, and resuspended in RNase‐free water. RNA quantification was performed using a Qubit fluorometer (Qiagen) and RNA quality was determined using a Fragment Analyzer (Advanced Analytical, Ames, IA). We set a RNA Quality Number (RQN) value cutoff of 5.7 for each sample.

### Library preparation and sequencing

Libraries were prepared using the QuantSeq 3′ mRNA‐Seq Library Prep kit (Lexogen, NH) following the manufacturer's instructions. Sequencing was performed with an Illumina NextSeq 500 (Illumina, San Diego, CA). Approximately, 400M single end, 75 bp reads were generated.

### Bioinformatics

Raw reads were aligned and converted into Ensembl IDs using STAR, which was also used to limit gene counts to protein coding genes. DESeq2, a package within R, was used to determine differential expression across groups. DAVID (NCBI, NIH) (da Huang et al. 2009a,b) and Biocarta were used to map enriched genes on a pathway diagram to determine clustering within specific signaling pathways. Cytoscape (version 3.2), an open source bioinformatics platform developed by the Institute of Systems Biology, Seattle,WA, was used to construct network diagrams and to illustrate clustering of the genes in our dataset within specific cell stress pathways (Shannon et al. 2003; Smoot et al. 2011). Gray nodes provide a skeleton diagram of known cell stress pathways. Round green nodes indicate genes from our dataset with *P*_adj_ < 0.05, and the size of the node indicates the degree of significance of upregulation (the diameter of the circle was determined by taking the log of the reciprocal of the *P* value.) Nodes with small green diamonds indicate stress‐responsive genes that were upregulated, but did not make the *P*_adj_ < 0.05 cutoff.

### Histology

Tissues sections were processed and analyzed as previously described (Dreyer et al. 2006) with modifications based on the methods from (Fry et al. 2014, 2015). Muscle biopsies from TKA patients were frozen in isopentane and stored at −80°C. Of the 13 subjects in the study, tissue for histology was obtained from 10 (eight females, two males; average age = 68.1 years). Sections (7 *μ*m) were cut using a Leica Cryostat (CM1850UV) set to −20°C. Following treatment with acetone for 5 min, the sections were blocked in PBS for one hour and primary antibody was added overnight at 4°C. Samples were incubated in secondary antibodies and sections were imaged on a Leica Epifluorescence microscope (Leica DM4000B). Primary antibodies used included anti‐laminin (DAKO, Carpinteria, CA, USA; rabbit) (1:2000), anti‐myosin heavy chain I (BA‐D5; mouse IgG2b) (1:50), anti‐myosin heavy chain IIa (SC‐71; mouse IgG1) (1:500), and anti‐myosin heavy chain IIx (6H1; mouse IgM) (1:100) (Developmental Studies Hybridoma Bank (DSHB), Ames, IA. Secondary antibodies included Alexa Fluor‐labeled mouse IgG2b (1:500), Alexa Fluor‐labeled mouse IgG1 (1:500), Alexa Fluor‐labeled mouse IgM (1:500), and Cy3‐labeled anti‐rabbit (1:200) (Molecular Probes, Eugene, OR).

### Cross‐sectional area analysis

Images were captured and analyzed as previously described (Dreyer et al. 2006). Measurements of cross‐sectional area were performed using MetaMorph. Muscle fiber boundaries were visualized using laminin immunofluorescence and traced. Cross‐section area was obtained for each fiber type based on immunofluorescence and calculated based on computer‐assisted software provided by Leica and based on the number of pixels within each boundary for each cell type. Statistical analysis of CSA data was performed using paired t‐test comparing pre‐ and post‐TKA fibers by type. Differences between means were considered significant at *P *≤* *0.05.

## Results

### Muscle cross‐sectional area

**Figure 1:**
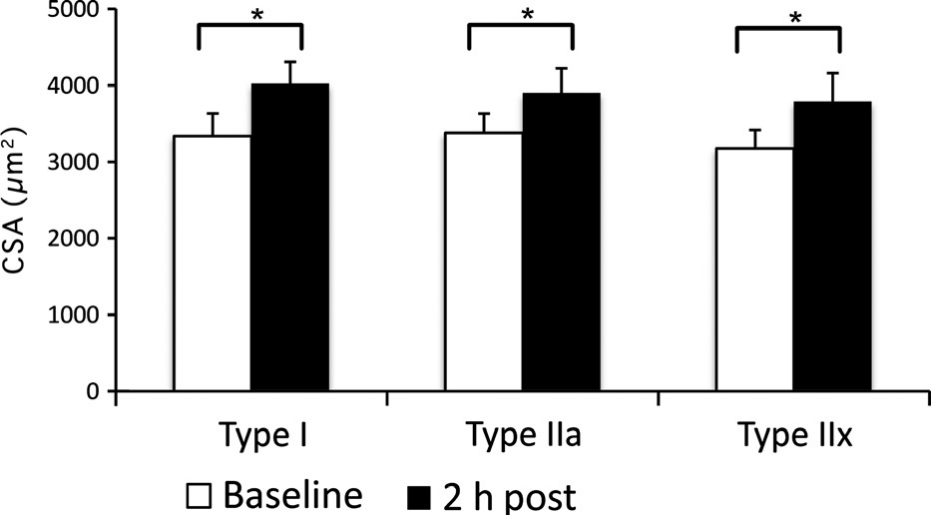
Muscle cross‐section analysis reveals increase in area at 2 h post. Histogram showing increase in cross‐sectional area of muscle fibers following TKA with tourniquet in each of the fiber types. *Y*‐axis shows *μ*m^2^. *Indicates statistical significance (*P* < 0.05) based on a Student's *t*‐test. Error bars indicate standard error (SE).

To characterize the possible tissue changes that occur after TKA with tourniquet, we analyzed histological sections of biopsies taken from patients before surgery and approximately 2 h after surgery. To determine the cross‐sectional area of muscle fibers, we labeled the tissue with anti‐laminin, an extracellular matrix component. Furthermore, we used muscle fiber type‐specific markers to distinguish between Type 1, Type 2a, and Type 2x muscle fibers. We found that the mean cross‐sectional area increased significantly following surgery for all types (Fig. 1).

### Gene expression

To gain a deeper understanding of the signaling mechanisms that may underlie the morphological changes in muscle and/or may lead to pathology and adverse outcomes following surgery, we performed NextSeq gene expression analysis to determine differentially regulated genes following surgery and tourniquet. Our analysis revealed 72 genes with significant differential expression (*P*_adj_ < 0.05). To understand the altered expression landscape, we mapped a large number of the differentially expressed genes on established, annotated pathways (such as KEGG and Biocarta), and for less well‐known genes that were not present in these established pathway maps, we searched the literature to determine putative function.

**Figure 2:**
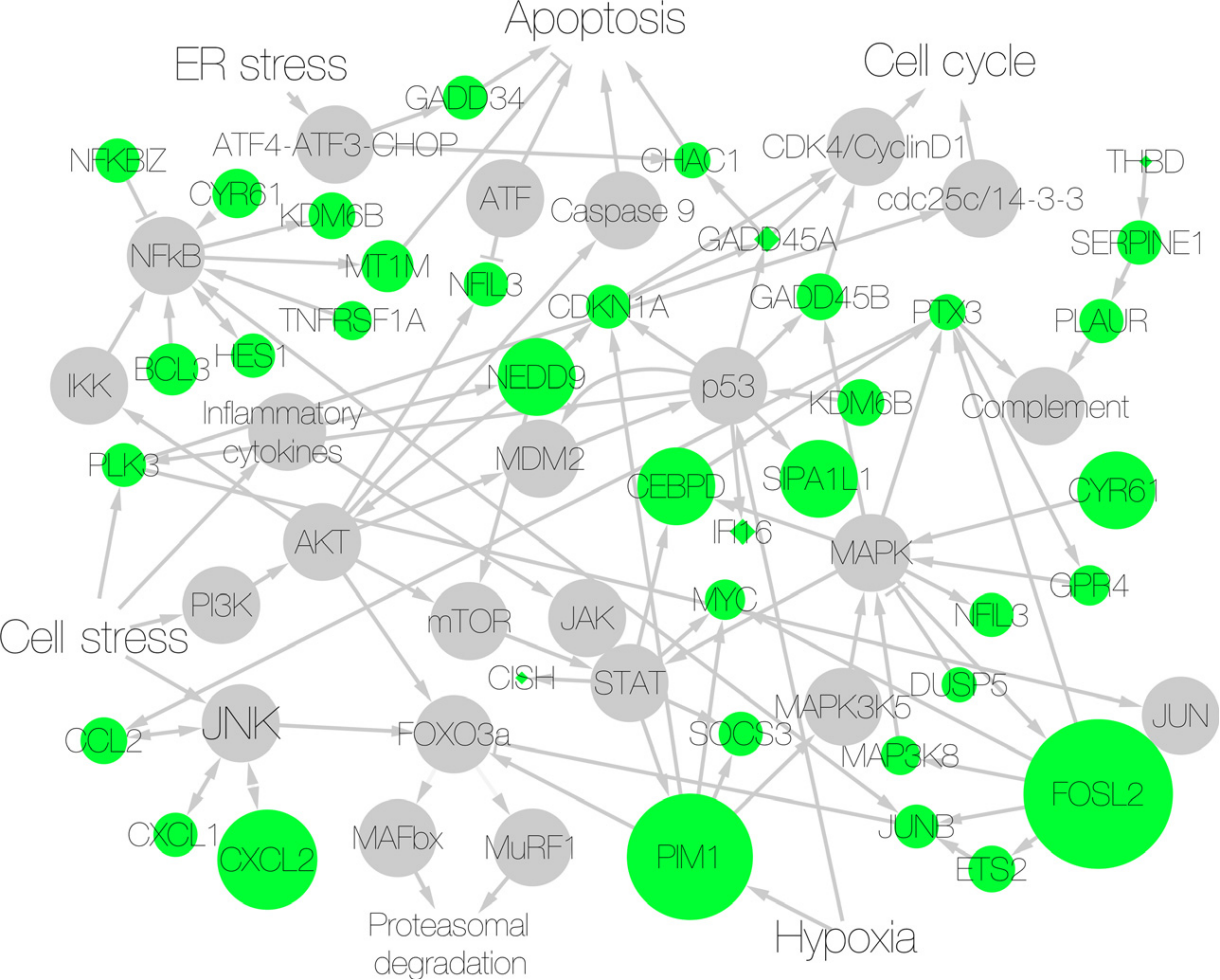
Cell stress pathways upregulated in TKA with tourniquet patients. Gene regulatory network diagram showing upregulated genes (green nodes) from our dataset overlaid on cell stress pathways. Size of green nodes represents the degree of upregulation. Gray nodes provide a skeleton diagram of known pathways to give context to the genes that were upregulated, but were not themselves represented in the list of differentially expressed genes. Arrows indicate a positive, directional interaction. Hammerheads indicate a negative, directional interaction.

A substantial subset (33 out of 72) of the genes mapped to cell stress pathways. To illustrate the interconnectedness of these gene regulatory networks, we constructed simplified network diagrams from established pathways using Cytoscape and overlaid genes from our dataset onto the maps. The gene regulatory networks for cell stress are highly interconnected and complex, and the diagram presented in no way is meant to represent the entire universe of genes involved in response to stress (Fig. 2). However, a diagram including every player would be impossible to visualize, so we have provided a simplified version.

Cell stress pathways that were found to be altered included JAK‐STAT, p53, JNK, NFkB, Akt, and MAPK pathways (Table 1). Processes that are induced during cell stress include apoptosis, cell cycle regulation, and complement activation and each of these processes have been shown to be influenced by genes in our dataset (Table 1). Furthermore, many of the genes in the dataset are known to be responsive to oxidative stress, hypoxia, ultraviolet (UV), and ionizing radiation (IR) (Table 1).

**Table 1:** Annotated list of genes upregulated in TKA with tourniquet

Gene	*P*_adj_	Pathway/Function
Cell stess‐‐MAPK		
FOSL2	1.68E‐06	Transcriptio factor activated by MAPK, oxidative stress
CEBPD	0.0116525	bZIP transcriptio factor involved in inflammation; apoptosis
CYR61	0.0125334	ECM protein involved in integrin binding; MAPK, FAK, paxillin, and Rac signaling
GADD45B	0.0157925	p38/JNK, MAPK inducible; apoptosis
GPR4	0.0262557	G‐protein coupled receptor; PTX sensitive; interacts with p38 MAPK
MAP3K8	0.030141	Stress activated; activates fos and jun
DUSP5	0.0427241	Negative regulator of MAPK
Cell stress‐‐JNK		
PIM1	1.36E‐05	Ser/thr kinase that binds STAT3/STAT5
CXCL2	0.0001392	Chemokine induced by NFkB and JNK
CCL2	0.0157925	NFkB inducible chemokine; chemotactic for monocytes, basophils
GADD45B	0.0157925	p38/JNK, MAPK inducible; apoptosis
CXCL1	0.0181886	Oxidative stress‐inducible cytokine
PIM1	1.36E‐05	Ser/thr kinase that binds STAT3/STAT5
Cell stress‐‐JAK‐STAT		
PIM1	1.36E‐05	Ser/thr kinase that binds STAT3/STAT5
CEBPD	0.0116525	bZIP transcription factor induces inflammation; apoptosis
CISH	0.0173316	SH2‐domain protein; STAT inhibition, cytokine‐inducible negative regulators of cytokine signaling
SOCS3	0.0181886	Negative regulation of cytokine signaling; STAT inhibition
Cell stress‐‐NFkB		
CXCL2	0.0001392	NFkB inducible chemokine
BCL3	0.009318	B cell proto‐oncogene and transcription factor that interacts with NFkB, fos, jun
CCL2	0.0157925	NFkB inducible; chemotactic for monocytes, basophils
FRMD8	0.0157925	FERM domain containing cytoskeletal protein, interacts with iKB
MT1A	0.015942	Metalotheionein involved in zinc metalation of carbonic anhydrase
CXCL1	0.0181886	Oxidative stress inducible chemokine
NFKBIZ	0.0182906	Inhibitor of NFkB
HES1	0.019757	bHLH transcription factor that interacts with sirtuin
GPR56	0.0198688	G‐protein coupled receptor that interacts with NFkB, mTOR
TNFRSF1A	0.029797	TNF receptor, activates NFkB, anti‐apoptotic proteins BCL‐2 interacting
PTX3	0.0380791	Complement‐dependent inflammation; activated by NFkB, jun, fos
Cell stress‐‐Akt		
NEDD9	0.018291	Crk‐associated substrate family protein that interacts with T cell receptor interaction; Integrin to Akt signaling
NFIL3	0.018789	Transciption factor that regulates IL3 transcription
RGS16	0.0220731	Regulator of G protein signaling; blocks mTOR,PIP3, Akt autophagy suppression
Cell stress‐‐p53		
GADD45B	0.015792	p38/JNK, MAPK inducible; apoptosis
KDM6B	0.015792	Lysine‐specific histone demethylase that regulates Wnt signaling
PLK3	0.0181886	Polo‐like kinase that interacts with p53; cell cycle
CDKN1A	0.0181886	Cyclin‐dependent kinase inhibitor controlled by p53
SIPA1L1	0.0377431	p53 degration
Cell cycle		
ETS2	0.0157925	ETS transcription factor interacts with c‐jun, CDK10
MYC	0.0279383	Nuclear protein; cell cycle, apoptosis, cell transformation
Cholesterol regulation		
CH25H	0.0064543	Cholesterol hydroxylase cleaves cholesterol in ER; IFN inducible
LDLR	0.0157925	Mediates endocytosis of LDL
Complement and coagulation		
HBB	0.0181886	Interacts with hemoglobin, alpha 1
SERPINE1	0.0194836	Serine protease that regulates fibrinolysis
PTX3	0.0380791	Complement‐dependent inflammation; activated by NFkB, jun, fos
Early response genes		
APOLD1	0.0157925	Early response endothelial apolipoprotein
ZFP36L1	0.0181886	Early response gene; zinf finger transcription factor
Glucose regulation		
SLC2A3	5.548E‐05	Glucose transporter involved in glucose regulation
PFKFB3	0.0004144	Glucose regulation, regulates CDK, cell cycle
Hypoxia/Oxidative stress inducible		
FOSL2	1.68E‐06	Transcription factor involved in MAPK
MAFF	0.0055774	bZIP transcription factor; binds NRF2 transcription factor for nuclear transport, competes with FOS for antioxidant response element binding
MT1X	0.0095178	Metallothionein
MT2A	0.0157925	Metallothionein; interacts with PD1
CXCL1	0.0181886	Oxidative stress inducible cytokine
MAT2A	0.0181886	Hypoxia‐induced, HIF‐dependent methionine adenosyltransferase II, alpha
RASD1	0.0220731	Oxidative stress inducible small GTPase involved in iron uptake regulation
PDE4B	0.0256044	Hypoxia inducible cAMP specific phosphodiesterase
MAP3K8	0.030141	Stress‐activated kinase; activates fos and jun
TIPARP	0.0370331	Oxidative stress inducible poly(ADP‐ribose) polymerase
CHAC1	0.0392172	Oxidative stress; degrades glutathione, apoptosis‐related transcription factors regulate
Immune cell regulation		
EGR3	0.0093178	Transcription factor that controls B and T cell proliferation
ZC3H12A	0.0157925	RNase involved in immune response, Toll‐like receptor responsive; mRNA decay
NEDD9	0.0182906	Crk‐associated substrate family; T cell receptor interaction; Integrin to Akt signaling
MMP19	0.0220731	Metalloproteinase; Th cells surface marker
CD83	0.0297967	T cell activation
TNFRSF1A	0.0297967	TNF receptor, activates NFkB, anti‐apoptotic proteins BCL‐2 interacting
SERPINB9	0.0440829	Serine protease inhibitor protects mast cells against apoptosis
Wnt signaling		
CSRNP1	0.0116525	Nuclear protein induced by axin1 (Wnt); induces apoptosis
KDM6B	0.0157925	Lysine‐specific histone demethylase that regulates Wnt signaling
GJA1	0.0181886	Gap junction protein that regulates cell communication; connexin; Wnt target gene

Besides the pathways mapped above, genes from our dataset clustered to several other categories. These included glucose regulation, early response genes, genes that regulate immune cell differentiation and activation, and regulators of the Wnt signaling pathway (Table 1).

A complete set of genes that were upregulated is presented in Table S1.

## Discussion

During TKA, muscle cells are subjected to prolonged oxygen deficiency that alters cell metabolism in order to reduce energy (ATP) demand and maintain cell homeostasis until blood flow is restored. We have recently reported on alterations in cell signaling pathways indicating a decrease in cap‐dependent translation initiation and elongation (Ratchford et al. 2012), an upregulation of stress‐activated protein kinases (JNK) and catabolic activation involving MuRF1 and MAFbx (Bailey et al. 2012), as well as induction of all three branches of the unfolded protein response (UPR) occurring due to endoplasmic reticulum (ER) stress. Our objective with this study was to obtain biopsy tissue at a later time point (2–3 h posttourniquet let down) in order to extend chronologically the effects of TKA on muscle cell metabolism in older adults during and immediately after surgery.

Our previous studies demonstrated that anabolic signaling was downregulated, presumably to conserve ATP for cellular processes critical to maintaining viability until blood flow and proper oxygen concentration are restored. Further, we have shown that significant cell and ER stress is occurring that may lead to apoptosis or overt cell death. As such, our findings that all three types of muscle cells (Type I, IIa, and IIx) become swollen, as measured by an increase in CSA, within 3 h of surgery, suggest energy levels failed to meet the demands of the Na/K pump and cellular osmolarity was not maintained. The increase in area can be explained by alterations in osmotic gradients, which favor an inward deflection of water from the extracellular space into the cytosol.

To gain a greater understanding of the tissue level changes observed and to detect underlying molecular changes that may contribute to muscle atrophy, we analyzed gene expression profiles following surgery with tourniquet. In fact, our NextSeq data revealed the activation of several cell stress‐related pathways including MAPK, JNK, JAK‐STAT, NFkB, Akt, and p53. Older subjects are, therefore, clearly capable of mounting a significant stress response following surgery. Although this response certainly plays a short‐term protective role following surgery, long‐term activation of this response can contribute to muscle atrophy. For example, JUNB and PIM1, two cell stress‐responsive genes upregulated in our study, interact with FOXO3a, a key component of the proteasomal degradation pathway and thus a potential factor in muscle atrophy in our study population. Furthermore, AKT and JNK also influence FOXO3a activity, and are regulated by factors in our dataset such as the inflammatory cytokines CXCL2, CXCL1, and CCL2. In addition, MuRF1, a key component of the proteasomal degradation pathway, is regulated by NFkB (in addition to FOXO3a), which is regulated by several molecules in our dataset including NFKBIZ, CYR61, KDM6B, MT1M, TNFRSF1A, HES1, and BCL3. Therefore, our gene expression analysis shows that TKA with tourniquet induces expression of the molecular components of muscle atrophy.

A hallmark of ischemia reperfusion injury is complement activation. Ischemia alone does not cause visible injury at the tissue level; however, it is thought that underlying molecular changes occur during ischemia that prime the tissue for injury following reperfusion. Although the full repertoire of factors that underlie these changes has yet to be characterized, the recruitment of IgM and associated complement factors is central to the process. While changes in tissue may elude visual detection, underlying invisible molecular signatures of these processes can be deduced by measuring gene expression with next generation sequencing methods. Our NextSeq data from patients following TKA with tourniquet revealed that genes related to complement activation such as HBB, SERPINE1, and PTX3 were upregulated, suggesting that the complement system is being recruited in these subjects. Furthermore, it is thought that oxidative stress contributes to complement activation, and our data set included several genes related to oxidative stress, most notably FOSL2, MAFF, CXCL1, and MAT2a. Therefore, we provide evidence that TKA with tourniquet induces changes in gene expression consistent with ischemia reperfusion injury.

A limitation of this study was that our clinical population did not include patients that received surgery without tourniquet or subjects who were administered tourniquet, but did not receive surgery. Thus, we cannot rule out that surgery was a significant or the sole contributor to changes in gene expression independent of tourniquet. However, the transcriptional response profile we observe is consistent with ischemia reperfusion injury, a response tourniquet application would logically be thought to induce. For this reason, although further studies are needed to tease apart the individual contributions of tourniquet and surgery, we feel these results are consistent with our conclusion that tourniquet application may prime older patients for ischemia reperfusion injury.

In conclusion, while further work is needed in order to measure the changes at later time points as well as following an anabolic stimulus, such as essential amino acid ingestion (Dreyer et al. 2008), we interpret the current findings as suggesting that tourniquet use may initiate proximate signals that initiate a cascade of events leading to the muscle loss that is evident within 2 weeks of surgery (Dreyer et al. 2013).

## Conclusions

Within the next two decades, the total number of TKAs performed annually in the U.S., is projected to increase to 3.5 million (Kurtz et al. 2011). Tourniquet is used in the overwhelming majority of TKA surgeries; however, our data suggest that tourniquet use may lead to tissue damage. Furthermore, we show that TKA patients initiate transcription of IR‐injury‐related genes, suggesting that tourniquet may induce underlying changes that may prime the tissue for reperfusion injury and potential muscle loss. While TKA has proven to be an effective surgical remediation for chronic knee pain associated with OA, several studies have suggested that there is a long‐term inability to regain muscle mass in older adults following TKA (Finch et al. 1998; Walsh et al. 1998; Mizner and Snyder‐Mackler 2005; Mizner et al. 2005a *,* 2005b; Yoshida et al. 2008) and that atrophy is the greatest contributor to functional mobility impairments (Meier et al. 2008, 2009). As such, insight into proximate causes of atrophy following TKA, which occurs at a rate of 1% per day for 2 weeks after surgery (Dreyer et al. 2013), are warranted in view of the fact that current strategy exchanges OA‐induced chronic knee pain with muscle loss. Muscle atrophy following surgeries in older adults is likely permanent and has the added complication of accelerating sarcopenia. Sarcopenia progresses normally at a rate of 1% per year in older adults (Dreyer and Volpi 2005), and in the U.S. in 2000 was estimated to cost $18.5 billion (Janssen et al. 2004). Reducing the prevalence of sarcopenia by 10% could save $1.1 billion/year (Janssen et al. 2004). Thus, identifying factors that may limit loss, such as attenuating IR‐injury associated with tourniquet use, is warranted to ensure the best clinical outcome for patients. While there is no question that eliminating knee pain is the most appropriate course of action, further research is needed in order to promote muscle recovery or, better yet, prevent loss during this critical time so that chronic mobility impairments are reduced or eliminated.

## Data Availability Statement

The data discussed in this publication have been deposited in NCBI's Gene Expression Omnibus (Edgar et al. 2002; Barrett et al. 2013) and are accessible through GEO Series accession number GSE75432 (https://www.ncbi.nlm.nih.gov/geo/query/acc.cgi?acc=GSE75432).

## Conflict of Interest

None declared.

## Supporting information




**Table S1.** Complete list of genes upregulated in TKA with tourniquet.
