# Supplementary material for: Transcriptional profiling and muscle cross‐section analysis reveal signs of ischemia reperfusion injury following total knee arthroplasty with tourniquet
Source: Physiol Rep. 2016 Jan 5;4(1):e12671. doi: 10.14814/phy2.12671 (PMC4760409; doi:10.14814/phy2.12671)
Supplement: Supplementary file 2 [file PHY2-4-e12671-s002.docx]

Table S1. Complete list of genes upregulated in TKA with tourniquet.
